# A Pilot Study of Interactive-Video Games in People with Mild Cognitive Impairment

**DOI:** 10.3390/ijerph19063536

**Published:** 2022-03-16

**Authors:** Yu-Fang Lin, Megan F. Liu, Mu-Hsing Ho, Yen-Kuang Lin, Yu-Ling Hsiao, Ming-Hsu Wang, Chia-Chi Chang, Jed Montayre

**Affiliations:** 1 School of Nursing, College of Nursing, Taipei Medical University, Taipei 11031, Taiwan; vichy@tmu.edu.tw; 2 School of Gerontology Health Management, College of Nursing, Taipei Medical University, Taipei 11031, Taiwan; fangliu@tmu.edu.tw; 3 School of Nursing, LKS Faculty of Medicine, The University of Hong Kong, Pokfulam, Hong Kong; mhbho@hku.hk; 4 Graduate Institute of Athletics and Coaching Science, College of Athletics, National Taiwan Sport University, Taoyuan 33301, Taiwan; robbinlin@ntsu.edu.tw; 5 Department of Nursing, College of Medicine, Fu Jen Catholic University, New Taipei City 242062, Taiwan; 125602@mail.fju.edu.tw; 6 Center for General Education, Taipei Medical University, Taipei 11031, Taiwan; mattwang@tmu.edu.tw; 7 College of Interdisciplinary Studies, Taipei Medical University, Taipei 11031, Taiwan; 8 School of Nursing and Midwifery, Western Sydney University, Sydney, NSW 2560, Australia; j.montayre@westernsydney.edu.au

**Keywords:** cognitive dysfunction, computer-assisted instruction, feasibility studies, independent living, video games

## Abstract

Early preventive strategies for improving cognitive function are crucial for people with mild cognitive impairment (MCI). Cognitive training exercises may improve cognitive functioning. However, there was limited evidence from training programs that combined cognitive-specific and physical activities, particularly in using interactive video games as interventions. This study aimed to evaluate the feasibility and effects of the interactive-video games on cognitive function, physical function, mood status and quality of life in community-dwelling people with MCI. A quasi-experimental study was undertaken. Participants in the intervention group received 60 min group-based training program once per week for 12 weeks. A generalised estimating equation (GEE) was used to examine the main effect, interactions and changes in outcomes over time. Sixteen participants completed the trial with eight in the intervention group and eight in the comparison group. The tolerable acceptance rate, perfect attendance rate, high satisfaction with the training content, and no injuries or falls demonstrated the feasibility of this program. The scores of cognitive function increased in both groups and the interaction between time and groups were significant over 12 weeks of training (*p* < 0.05). As the result, we determined that interactive-video games can be a safe, feasible, enjoyable intervention and user-friendly among people with MCI in community settings.

## 1. Introduction

The number of older people living with dementia is exponentially increasing [1]. According to the World Health Organisation, around 50 million people worldwide have dementia, with nearly 10 million new cases annually [2]. People living with dementia face several challenges, which impact not only on their own quality of life but also that of their primary caregivers [1,2]. Moreover, the enormous health expenditures on dementia care pose concerns and serious economic implications relating to health service delivery [2,3]. Despite the phenomenal increase in dementia cases globally, its incidence can be prevented or slowed down [1]. The recent focus on dementia research was on risk factors and at pre-diagnosis level. One of these studies has given closer attention to mild cognitive impairment as a pre-cursor of dementia [4,5].

Mild cognitive impairment (MCI) is a transition stage between normal ageing and dementia [6], more than 25% of people with MCI progress into dementia within five years [7]. Early preventive strategies for improving cognitive function are crucial for people with MCI, it holds the key towards positive public health and economic outcomes. Non-pharmacological interventions may benefit individuals by preventing or postponing the progression from MCI to dementia.

Cognitive training exercises, in general, may improve or optimise cognitive functioning and quality of life [8,9]. Moreover, combined cognitive and physical training resulted in positive effects on physical functioning and depression [9,10]. With the increasing development of science and technology, the application of computerised software, video games, and virtual reality platforms have become more popular in sports, education, and health disciplines [11,12]. Previous studies showed significant effects on cognitive function and psychosocial function by using computerised cognitive training with/without motor activity in people with MCI [13,14]. According the review of Gates et al. (2019), computerised cognitive training may improve the performance of global cognition, episodic memory and working memory. However, the evidence was limited and it was difficult to combine cognitive-specific and physical activity computer programs [15].

Participation and higher adherence to cognitive training program resulted in positive improvement in cognitive function [16]. Motivation to participate in cognitive training programs is key to successful implementation, which is highly dependent on the profile of participants and types of tools or training programs suitable for them. A growing body of evidence applied convenient commercial devices, such as the Nintendo Wii and Xbox Kinect, for gait/balance exercises, rehabilitation, and cognitive training for individuals who had a stroke, Parkinson’s disease, and cognitive impairment [17,18,19]. Interactive-video games have recently become popular since they are enjoyable and cost-effective [9,15]. However, most commercial products were designed for entertainment purposes and have low user-adaptability.

A recent Cochrane review on computerized training programs for MCI revealed that although positive effect and utility of computerized interventions in MCI have been established, the majority of these studies undertaken require a more robust methodological research protocol for outcomes to be conclusive [15]. Moreover, previous studies did not combine interventions or measured outcomes pertaining to whole body movement and function [15]. A recent Cochrane review suggested the need to further investigate on computerised cognitive training among MCI populations. This pilot study aimed to examine the feasibility (including acceptability, attendance, satisfaction with the training content and safety) as well as the effects of implementing a non-pharmacological approach using interactive-video games on cognitive function, physical function, mood status and quality of life in community-dwelling people with MCI. We hypothesised that interactive-video games program is a feasible intervention to maintain the cognitive function, physical function, mood status, and quality of life in people with MCI.

## 2. Materials and Methods

### 2.1. Design

This quasi-experimental study was undertaken in Northern Taiwan. Participants were recruited from community care centres and churches, as they participate in organised community activities (i.e., singing class, baking class) or the regular group activity (e.g., bible reading, singing hymns, and so on) in the church once a week. This study followed the Transparent Reporting of Evaluations with Non-randomised Designs (TREND) statement (Supplementary Figure S1).

### 2.2. Settings and Samples

Purposive sampling was used with the inclusion criteria of (1) aged ≥60, due to more than half of this population age group with MCI progress to dementia within five years [20]; (2) assessed using the Short Portable Mental State Questionnaire (SPMSQ) with scores between five and eight points [21]; (3) had no sensory deficits and can communicate or interact with research assistants. Exclusion criteria were: (1) had activity restrictions from physician; and (2) had unstable disease progression which could affect their participation, for example, severe depression. The operational definition of the people with MCI was participants with SPMSQ scores between five and eight points assessed by the primary investigator.

### 2.3. Ethical Considerations

This study was approved by Taipei Medical University Joint Institutional Review Board (TMU-JIRB No.: 201307025) for studies involving humans. Written informed consent was obtained from participants and their proxies. Participants were informed that they have a possibility to drop out of the study at any time.

### 2.4. Procedure

After the baseline assessment, the participants from the same community care centre or church were conveniently assigned to the same group (either intervention or comparison group) to prevent possible experimental contamination. To avoid long waiting time, two to four participants were in one intervention group. Participants in the intervention group received 60 min interactive-video games once a week under supervision by primary investigator for 12 weeks, and the comparison group kept their usual activities without any intervention. Outcome measures were assessed at baseline, 4th, 8th, and 12th weeks (the end of training period). In addition, we checked vital signs before the commencement of the intervention by the primary investigator (i.e., blood pressure greater than 160/100 mmHg should seek medical assistance to take Senior Functional Test [SFT]), via pulse oximetry technology of all participants, and carried out five to eight minutes’ warm-up activity before taking SFT, unipedal stance test and intervention for safety consideration.

### 2.5. Intervention Program

We utilised interactive-video games called “Xavix Hot Plus” (Hot-plus, SSD Co. Ltd., Shiga, Japan), which was designed specifically for rehabilitation and reported high participant motivation and enjoyment while playing [22,23]. It comprised five domains, including memory, visual reception, concentration, executive function, and social interaction, classified by this software development company [24]. Each domain consisted of multiple games that involve the application of psychomotor skills, such as hand-eye coordination, reckoning by time, and accurate control of three-dimensional space by the upper or lower limbs [22,23]. Each interactive-video game was chosen from different domains, one game for one domain. Each session contained five different games (domains), and each game played three formal trials, then, switched to the next game. Participants performed the same interactive-video games for four weeks and then moved on to the next session (see Table 1 and Supplementary Material) [22]. Primary investigator encouraged participants and selected the level of difficulty by their reaction in response to the combination of limited cognitive and physical activity computer programs. To maintain motivation, the types of interactive-video games were changed every four weeks. The games had different levels of difficulty for single or multiple players (details are shown in Table 1).

### 2.6. Treatment Fidelity

The treatment fidelity was achieved by following the guidelines [25]. The primary investigator was trained by a product manager to ensure that the procedure was performed correctly. The primary investigator observed their functional performance and reaction abilities, then selected the appropriate levels of difficulty. The participants were allowed two practice attempts when they started a new session. Participants in comparison group were not provided any intervention but only followed by phone interview for data collection. To ensure the adherence to treatment protocol, all participants received a booklet contained all of the records and appointments.

Each interactive-video game had a brief instruction. The primary investigator explained the instruction and demonstrated to ensure the participants fully understood, and then repeated under supervision to confirm their practice was performed correctly.

### 2.7. Measurements

Feasibility and preliminary outcomes included: (a) acceptability: the acceptance rate was counted from the potential participants; (b) attendance: the attendance rate in the intervention group; (c) satisfaction with the training content: all participants in the intervention group rated, on a four-point Likert Scale (‘not at all’—to ‘very much’), their level of satisfaction with the training content, the overall feeling, the schedule arrangement, and the venue for the activity, and their favourite games; (d) adverse effects: recording the details of who, when, where, and how (i.e., any observed injuries or falls during the intervention); and (e) preliminary outcomes were measured by established good reliability and validity instruments (details in Table 2). All data collectors included four research assistants collected outcome measures after training, one research assistant with physical therapist background integrated and checked all collected data. Data collectors were blinded to the group allocation.

### 2.8. Data Analysis

The Mann-Whitney U test and Fisher’s exact test were used to compare the baseline differences due to non-normality of the data and small sample size categorical variables. Generalised Estimating Equation (GEE) was used to examine the main effects, time effects, and interactions in outcomes over time. We adopted the first-order autoregressive (AR-1) covariance as the working covariance structures in the GEE estimation of the primary outcome, and the exchangeable covariance as the working covariance structures in the GEE estimation of secondary outcomes. The dependent variable is assumed to follow a normal distribution. Then, GEE estimates of model parameters (B) are obtained using maximum likelihood estimation to form the equation. All analyses were carried out using SPSS Version 19.0 (IBM SPSS Statistics for Windows).

## 3. Results

### 3.1. Participant Characteristics

**Figure 1:**
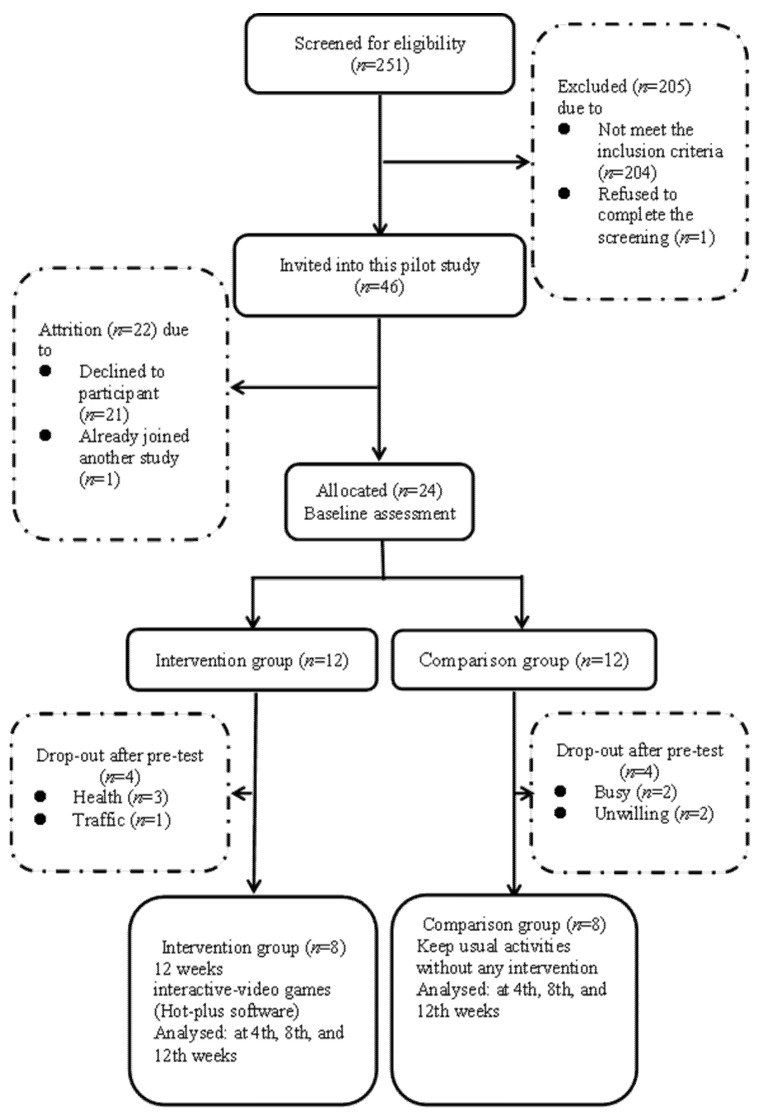
Flow diagram.

Of 251 potential participants screened, 46 were invited and 24 agreed to participate with 12 in each of the intervention and comparison groups at the time of recruitment. Sixteen participants were included in the final analyses. Four people in the intervention group dropped-out were not related to the treatment provided, one due to the traffic difficulty and three due to health issues such as receiving surgery and not feeling well when they were invited to receive the next session of the intervention. With the remaining eight participants in the intervention group as well as eight participants in the comparison group (Figure 1). There were non-significant differences between the two groups in demographics, health condition, fall information, and regular exercise at baseline data (Table 3). However, there were significant differences between the two groups in the Instrumental Activities of Daily Living Scale (IADL), the Geriatric Depression Scale Short Form (GDS-SF), and the five domains of the Euroqol Health utility score (EQ5D-Utility) at baseline (*p* = 0.027, 0.038, 0.045, respectively), others measures showed non-significant differences between the two groups.

### 3.2. Feasibility

Of the 251 eligible people screened, 205 (81.7%) did not meet the inclusion criteria and one refused to complete the screening. Forty-six potential participants were invited and 24 agreed to participate with 12 in each of the intervention and comparison groups at the time of recruitment (Figure 1). The acceptance rate was 52.2%. Besides, those participants (*n* = 8) who stopped attending sessions after baseline assessment or before the intervention were excluded. In the intervention group, three drop-outs were due to health problems (i.e., low back pain or surgery) and one was due to traffic consideration reduced the willingness for participation.

In the intervention group, the attendance rate of all participants remained was 100%. The majority of participants were “very” satisfied with the training content (M = 3.88, SD = 0.33), the overall feeling (M = 3.75, SD = 0.43), the schedule arrangement (M = 3.50, SD = 0.71), and the venue for the activity (M = 3.63, SD = 0.70) were all reported as satisfied.

### 3.3. Preliminary Outcomes

#### 3.3.1. Primary Outcome

**Figure 2:**
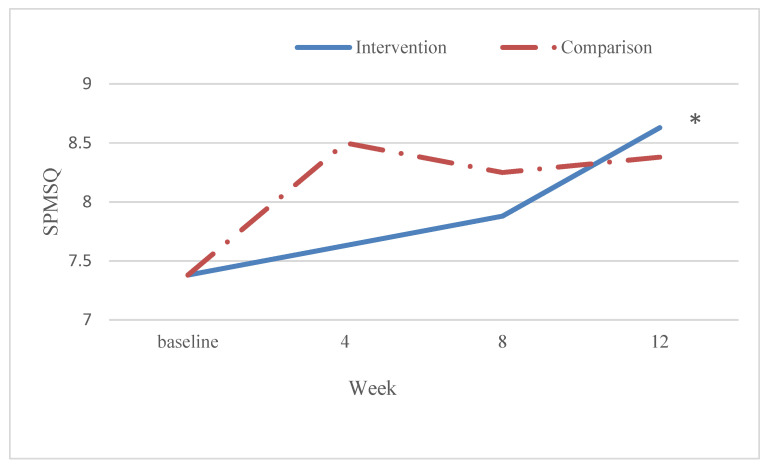
The mean plot for SPMSQ score between the intervention group and comparison group. SPMSQ, Short Portable Mental Status Questionnaire. * *p* < 0.05. Interaction between group and time.

We examined cognitive function by main effect (the difference between the intervention and the comparison group), time (the change over the study period of 12 weeks), and group-by-time interaction (group-by-time interaction refers to the change between these two groups over time) (Figure 2). The interaction between time and group was significant over 12 weeks of training (*p* < 0.05) (Table 4). Although the comparison group performed better at 4th and 8th weeks, the intervention group sustained and performed even better after the 12 weeks intervention. In particular, the mean score of the comparison group was increased from 7.38 to 8.38, while the mean score of the intervention group was improved from 7.38 to 8.63. According to the result, the improvement of the intervention group was better than the comparison group (Table 4).

#### 3.3.2. Secondary Outcome

Regarding physical function, there were significant differences between two groups in IADL (*p* = 0.027) at baseline but no significant trend difference (Table 4). In the sum of SFT, except for the Chair stand test and the Chair sit and reach test which had a significant trend difference between two groups by time effects (*p* = 0.019, 0.024, respectively). Others, had no significant trend difference by the main effect, time effect, and group-by-time interaction over time (Table 4).

Although the score of GDS-SF and the score of the EQ-5D utility had a significant difference between two groups (*p* = 0.038, 0.045, respectively) in the baseline, no significant difference over time were observed (Table 4).

## 4. Discussion

Our study showed significant improvements in the trend of cognitive function over time, and participants in the intervention group had higher satisfaction and attendance rate. In terms of feasibility, it is likely that individuals, particularly attendees are at risk of injuries (e.g., falls) from playing the interactive-video games [45]. With respect to adverse effects, no injuries or falls were observed during the intervention. Similar findings were noted in previous studies using interactive-video sports games (The Nintendo Wii™ games) for a duration in 90 min once per week for 24 weeks [46]. Keogh et al. (2014) also used the same interactive-video sports games (The Nintendo Wii™ games) for 34 older people living in the residential aged-care centre [9]. Participants in the intervention group selected the frequency, duration, and type of games by themselves. The average training time was 30 ± 24 min per week for eight weeks and found that had significantly improved in the upper body muscle strength and endurance, levels of physical activity, and psychological quality of life but not in the functional ability and dynamic balance [9]. However, in our study, regarding the non-significant results in agility, dynamic and static balance, and cardiovascular endurance, these functions might need to have longer time and higher frequency. Future research aims at investigating the effects of interactive-video games on these outcomes with a longer period of time is recommended.

To highlight the innovative training program of the combination of physical activity and computerised programs, Shatil (2013) also reported that either cognitive training alone or combined with physical activity could significantly improve cognitive function on hand-eye coordination, global visual memory (working memory and long-term memory), speed of information processing, visual scanning, and naming in healthy older people [47]. In this study, we used interactive-video games since they were designed for assisting the rehabilitation and physical training of patients. One study also used the same interactive-video games as intervention, including cognitive function as an outcome measurement. They implemented two types of games (i.e., grab coins and drum beat) once a week for continuous 10 weeks in nine older residents with mild to moderate dementia in a nursing home. Their findings demonstrated that the general cognitive function, visuospatial and constructive function, and behaviour of older adults were improved after the intervention but no significant while they did not mention ‘time duration’ in their intervention [22]. In our study, compared to the comparison group, the participants in the intervention group had a greatest improvement in the SPMSQ score was obtained between the eighth and twelfth measurement points. The interactive-video games that we used to involve in the application of psychomotor skills visual–spatial ability required the participants to have accurate control of three-dimensional space by their upper or lower limbs to get a higher score. The interactive-video game “Hide and Seek” was the only game which trained to distinguish spatial recognition tasks including spatial relationship and orientation. The spatial recognition task involved choosing the correct window where the child hides after the house rotating. The degree of the rotating was from 0 to 180. Since the visuo-spatial function may potentially be affected [48], the reaction in short time was difficult for older people with MCI. Furthermore, interactive-video games have the potential to increase the level of dopamine in the brain which may produce pleasure feelings [49]. Thus, the overall satisfaction of participants was high in our study. In response to the favourite games, the memory training types were the most disliked games since the participants felt they were challenging to remember.

Although the mean GDS-15 score for the intervention group was above 5 at baseline, the effect size was very small (*r* = −0.49). We used the GEE model which could adjust the effect of baseline differences between the intervention group and the comparison group. The mean score in the intervention group was decreased from baseline gradually (from 5.25 to 4.25) and a little increased at the 12th week (4.50). The mean score in the comparison group was a slightly increased from baseline to the 4th week (from 2.75 to 2.88) than decreased at the 8th week (2.00) and maintained until the 12th week. As age increased, the rate of inactivity rates among older people increased [50]. Older people who had a sedentary lifestyle had suffered depression more frequently [51]. We could see the improvement trend on the GDS score over time in both groups. Although it had no significant due to small sample size, it also benefited to the mood status of older people by encouraging them to go outside to participate in activities. Furthermore, the association between depressive symptoms and cognitive function is complex. Previous studies found that depressive symptoms increase the risk in older people with MCI, often progressing to dementia [52,53]. In our study, cognitive function improved in both groups and had a significant change over time (from baseline to the 12th week). Potter and Steffens (2007) suggested applying non-pharmacologic interventions to older people with depression and cognitive impairment should focus on increasing daily pleasant events. The interactive-video games what we used exactly in accordance with creating enjoyment and increasing the motivation to participate [54]. Future research can extend the sites to approach more eligible participants (e.g., the outpatient departments at hospitals). Also, to include more sites in the community can increase the generalizability of the study results.

### Limitations

Firstly, people living with MCI are a hard-to-reach group, particularly in the community settings. The small sample size and the nonrandomized design of this study may limit its generalisability. Thus, these results need to be interpreted with caution. Secondly, the training dosage, 60 min per week for 12 weeks, may not be enough to have a significant benefit on the cognitive function, physical function, mood status, and quality of life. Increasing the frequency of these interactive-video games participation per week or duration of the trial may reduce the acceptability and adherence to the intervention. Thirdly, conducting an interactive-video game for cognitive training was challenging with the target population, as many of them have limited technology literacy. Lastly, this study only examined the outcome measures within 12 weeks follow-up. Little is known about the long-term effects of these interactive-video games.

## 5. Conclusions

Our study revealed that the interactive-video games could be a safe, feasible, and enjoyable intervention among people with MCI in community settings. Interactive-video games might be potentially suitable for people with MCI to maintain their cognition. Interactive-video games are likely to maintain the cognitive function of people with MCI. Although the participants in the comparison group had better improvement in cognitive function in the early intervention weeks, the participants in the intervention group progressed steadily and caught up later. As for secondary outcomes, the participants in the comparison group had better performance in terms of physical function, mood status, and quality of life, and all outcomes were decreased except for the lower-body strength and balance of SFT (as well as mood status). On the other hand, outcomes in the intervention group were improved except IADL, eight-foot TUG of SFT, and EQ5D-VAS of the quality of life. It is also user-friendly to combine a cognitive training and physical activity by using the interactive-video games. Future studies can consider implementing the interactive-video games in memory training and further testing the effectiveness of the program.

## Figures and Tables

**Table 1:** Interactive-video games training program.

	Games	Session 1 (1–4 Weeks)	Session 2 (5–8 Weeks)	Session 3 (9–12 Weeks)
Training Domains				
Memory		Name: *Step on Numbers* ^a^ Brief introduction: Step on a sensed pad in a forward or backward order by the changing number from one to twenty or one to thirty on the screen and scores depend on how fast the participant finishes. Psychomotor skills: Leg-eye coordination, lower-limb ROM, strength, attention.	Name: *Pair the Figure Cards* ^a^ Brief introduction: Flip over virtual cards and pair them with the same figures and scores depend on how fast the participant finishes. Psychomotor skills: Phalanges ROM, hand grasping, attention.	Name: *Hide & Seek* ^c^ Brief introduction: Find out the correct window where the child hides with or without the house rotating and scores depend on how fast and correctly the participant finishes. Psychomotor skills: Phalanges ROM, hand grasping, visual–spatial ability, attention.
Visual reception		Name: *Open the Door* ^a^ Brief introduction: Open the randomized one side or two sides’ virtual doors in 60 s. Psychomotor skills: Hand-eye coordination, upper-limb ROM, strength, endurance, attention.	Name: *Throw the Circles* ^a^ Brief introduction: Virtual ring toss, mimic throwing a sensed ring to land on virtual targets in 60 s. Psychomotor skills: Hand-eye coordination, upper-limb strength, visual–spatial ability, sensorimotor control.	Name: *Archery* ^a^ Brief introduction: Step on a sensed pad to shoot a virtual arrow at a virtual target in 60 s. Psychomotor skills: Leg-eye coordination, lower-limb ROM, strength, endurance, visual-spatial ability, attention.
Concentration		Name: *Play Bowling* ^a^ Brief introduction: Knock down all bowling pins with a sensed bowling ball in 60 s; different colour pins have different scores. Psychomotor skills: Upper-limb ROM, visual-spatial ability, sensorimotor control, attention.	Name: *Golf Greens* ^a^ Brief introduction: Compete in a virtual golf tournament with a sensed golf club. Psychomotor skills: Upper-limb ROM, hand function, trunk rotation, sensorimotor control, visual–spatial ability.	Name: *Climb Ladders* ^a^ Brief introduction: Keeping either one hand or two hands on a virtual ladder and count how high the participant can climb up in 60 s. Psychomotor skills: Hand-eye coordination, upper-limb ROM, strength, endurance, hand function, visual–spatial ability, attention.
Executive function		Name: *Shoot Basketball* ^a^ Brief introduction: Use a sensed bar to shoot randomized virtual red or white basketballs into the baskets with the same colour on the two sides in 60 s. Psychomotor skills: Hand-eye coordination, upper-limb ROM, strength, sensorimotor control.	Name: *Step on Hamsters* ^b^ Brief introduction: Step on a sensed pad corresponding to the virtual holes and scores according to the number of virtual hamsters stepping in 60 s. Psychomotor skills: Leg-eye coordination, lower-limb ROM, strength, endurance, attention.	Name: *Follow the Circles* ^a^ Brief introduction: Trace virtual circles in 60 s. Psychomotor skills: Hand-eye coordination, upper-limb ROM, strength, hand dexterity, attention.
Social interaction		Name: *Play a Drum* ^a^ Brief introduction: Step on a sensed pad with the tempo of the chosen music. Psychomotor skills: Leg-eye coordination, lower-limb ROM, strength, endurance, attention.	Name: *Bowling Tournament* ^b^ Brief introduction: Compete in a virtual bowling tournament with a sensed bowling ball. Psychomotor skills: Hand-eye coordination, upper-limb ROM, visual–spatial ability, sensorimotor control.	Name: *Paper Sumo* ^b^ Brief introduction: Step on a sensed pad to force a virtual or another opponent to the ground or pushes him out of the ring. Psychomotor skills: Leg-eye coordination, lower-limb ROM, strength, endurance.

ROM, range of motion; ^a^ Three levels of difficulty with single player; ^b^ Three levels of difficulty with mutiple players; ^c^ Two levels of difficulty with single player. (Supplementary Figure S1).

**Table 2:** Description of the preliminary outcomes instruments.

Name	Domains	Item	Measures and Scoring	Reliability	Validity
**Primary outcome**					
*Cognitive function*					
SPMSQ [26]	Orientation, working memory, and calculation [27].	Total: 10	Maximum score: 10 It was considered to indicate cognitive impairment if the number of correct answers was fewer than 8 points [26,27,28]^.^	Internal consistency: 0.98 [29].	Correlates with the MMSE, *r*: 0.81 [30].
**Secondary outcome**					
*Physical function*					
IADL [31]	Shopping, transportation, meal preparation, ordinary housework, doing laundry, medications, phone use, and managing finances.	Total: 8	Maximum score: 8 Lower scores indicating greater difficulty in performing instrumental activities [31,32].	Test-retest reliability: 0.93 [33].	Correlates with the Physical Classification (PC) scale, the MSQ, *p* < 0.01 [31].
SFT [34]				Test-retest reliability: 0.89~0.95 [34].	Good validity [34].
	Chair stand test	The lower-body strength	Total number of repetitions completed in 30 s.	Test-retest reliability: 0.89	Correlates with 1 RM leg press strength, the criterion validity, *r* = 0.77, 95% CI = 0.63~0.88 [34,35].
	Eight-foot TUG	Dynamic balance and agility	Recorded the time in seconds. Get up from a chair, walk as quickly as possible around a cone and return to sit back down in the chair (8 feet, 2.44 m).	Test-retest reliability: 0.95	Correlates with TUG, the concurrent validity, *r* = 0.85~0.92, *p* < 0.001 [36].
	Chair sit and reach test	Hamstring flexibility	Number of CM from the tips of the middle fingers short of reaching to the top of the shoe (minus score), touched the toes (zero scores), or past the toes (plus score).	Test-retest reliability: 0.95	Correlates with goniometer-measured hamstring flexibility, the criterion validity, *r* = 0.76~0.81 [34].
	6MWT	Measuring cardiovascular endurance	The distance walked as quickly as possible for six minutes, without running or jogging. Two cones were placed at 15 m intervals.	Test-retest reliability: 0.94	Correlates with modified Balke treadmill protocol, the criterion validity, *r* = 0.71~0.82 [34].
Balance					
	UPST [37]	The static balance on the preferred leg.	Recorded the time in seconds with the eyes open. Higher value indicated the better function.	ICC: 0.86.	Correlates with Tinetti Balance Subscale, the concurrent validity, *r* = 0.57 [38].
*Mood status*					
GDS-SF [39]	Self-rating of symptoms of depression	Total: 15 “yes/no” questions. Answered positively indicated the presence of depression: 10 Answered negatively indicated depression: 5	Normal: 0–4 Mild: 5–9 Moderate to severe: ≥10 [40]	Cronbach α: 0.89 [41].	Correlates with the GDS long form, *r*: 0.93 [41].
*Quality of life*					
EQ5D-VAS [42]		Rated current health status in a 20-cm visual analogue scale (VAS)	Imaginable health state: Worst: 0 Best: 100	Cronbach’s α of the Taiwanese version: 0.70 [43].	Correlates with the SF-12 subscale, the concurrent validity, *r*: 0.45~0.49 [43].
EQ5D-Utility	Mobility, self-care, usual activities, pain/discomfort, and anxiety/depression	Coded as three levels: 1, no problem; 2, moderate problems; 3, extreme problems.	According to the Converted to a single summary utility score by TTO. Range: −0.67 to 1.00 by using the Taiwanese value set [44]. Closer to 1: the better health, Negative: worse than dead, 0.5: acceptable.	Cronbach’s α of the Taiwanese version: 0.51 [43].	Correlates with the SF-12 subscale, the concurrent validity, *r*: 0.42~0.53 [43].

SPMSQ, Short Portable Mental Status Questionnaire; IADL, Instrumental Activities of Daily Living Scale; MSQ, Mental Status Questionnaire; SFT, senior functional test; TUG, timed up and go test; 6MWT, 6-min walk test; UPST, timed unipedal stance test; GDS-SF, geriatric depression scale short form; EQ5D-VAS: the five domains of the Euroqol Health visual analogue scale (VAS); EQ-5D-Utility, the five domains of the Euroqol Health utility score. MMSE: Mini-Mental State Examination. ICC: The intraclass correlation coefficient. TTO: The time trade-off. SF-12: Short-Form 12 Health Survey.

**Table 3:** Participant characteristics in demographic data of both groups (N = 16).

Variables	Intervention Group (*n* = 8)	Comparison Group (*n* = 8)	
	*n* (%)	*n* (%)	*p*
**Demographic**			
*Age* (M *±* SD)	79.75 *±* 4.86	77.75 *±* 6.74	0.597
60~69 (y/o)	0 (0.0%)	1 (12.5%)	
70~79 (y/o)	3 (37.5%)	4 (50.0%)	
80~89 (y/o)	5 (62.5%)	3 (37.5%)	
*Gender*			1.000
Male	3 (37.5%)	3 (37.5%)	
Female	5 (62.5%)	5 (62.5%)	
*Education level*			0.608
≤Elementary school	4 (50.0%)	6 (75.0%)	
≥Junior High School	4 (50.0%)	2 (25.0%)	
**Health Conditions**			
*Amount of chronic health conditions*			0.282
≤1	1 (12.5%)	4 (50.0%)	
≥2	7 (87.5%)	4 (50.0%)	
**Fall Information**			
*Previous history of falls* (*yes*)	6 (75.0%)	3 (37.5%)	0.315
**Regular Exercise** (*yes*)	4 (50.0%)	7 (87.5%)	0.282
*Cognitive function*			
SPMSQ (M *±* SD)	7.38 *±* 0.74	7.38 ± 0.92	0.860
*Physical function*			
IADL (M *±* SD)	7.25 ± 1.04	8.00 ± 0.00	0.027
SFT			
Chair Stand Test (rep) (M *±* SD)	10.25 *± 5*.97	13.75 *± 2*.32	0.051
8-foot TUG (sec) (M *±* SD)	16.71 *±* 14.44	7.65 ± 1.78	0.172
Chair Sit and Reach Test (cm) (M *±* SD)	−1.04 *±* 10.00	8.06 ± 9.88	0.400
6MWT (m) (M *±* SD)	292.76 ± 192.40	355.38 ± 90.45	0.600
Balance			
UPST (sec) (M *±* SD)	4.60 ± 2.75	19.09 ± 18.36	0.059
*Mood status*			
GDS-SF (M *±* SD)	5.25 ± 2.77	2.75 ± 1.98	0.038
*Quality of life*			
EQ5D-VAS (M *±* SD)	64.04 ± 28.24	68.88 ± 23.69	0.713
EQ5D-Utility (M *±* SD)	0.70 ± 0.22	0.89 ± 0.16	0.045

SPMSQ, Short Portable Mental Status Questionnaire; IADL, Instrumental Activities of Daily Living Scale; SFT, senior functional test; TUG, timed up and go test; 6MWT, 6-min walk test; UPST, timed unipedal stance test; GDS-SF, geriatric depression scale short form. EQ5D-VAS, the five domains of the Euroqol Health visual analogue scale (VAS). EQ-5D-Utility, the five domains of the Euroqol Health utility score. The Mann-Whitney U test and Fisher’s exact test were used to compare the baseline differences due to non-normality of the data and small sample size categorical variables.

**Table 4:** GEE Models between groups for cognitive function, physical function, mood status, and quality of life by group, time, and group-by-time interaction: Baseline to 12 weeks.

Item	Group	Visit				Group	Time	Interaction
		Baseline	4	8	12	*p*	*p*	*p*
		M *±* SD	M ± SD	M ± SD	M ± SD			
**Cognitive function**								
*SPMSQ*	I	7.38 ± 0.74	7.63 ± 1.06	7.88 ± 1.46	8.63 ± 1.06	0.030	0.005	0.049
	C	7.38 ± 0.92	8.50 ± 1.07	8.25 ± 1.17	8.38 ± 1.41			
**Physical function**								
*IADL*	I	7.25 ± 1.04	7.63 ± 0.74	7.38 ± 1.41	7.13 ± 1.46	0.121	0.394	0.394
	C	8.00 ± 0.00	7.75 ± 0.46	7.63 ± 0.74	7.75 ± 0.71			
*SFT*								
Chair Stand Test (rep)	I	10.25 ± 5.97	10.00 ± 5.76	10.00 ± 6.76	11.50 ± 6.23	0.065	0.019	0.090
	C	13.75 ± 2.32	15.13 ± 2.42	15.13 ± 4.64	15.13 ± 2.10			
8-foot TUG (sec)	I	16.71 ± 14.44	19.42 ± 19.98	26.19 ± 36.07	23.07 ± 29.40	0.379	0.274	0.374
	C	7.65 ± 1.78	7.16 ± 1.96	7.97 ± 1.82	7.82 ± 1.88			
Chair Sit and Reach Test (cm)	I	−1.04 ± 10.00	1.19 ± 8.34	1.06 ± 7.03	−0.88 ± 7.77	0.701	0.024	0.863
	C	8.06 ± 9.88	6.38 ± 9.68	5.14 ± 11.20	4.56 ± 10.78			
6MWT (m)	I	292.76 ± 192.40	315.78 ± 207.00	289.85 ± 187.30	314.82 ± 201.91	0.129	0.922	0.488
	C	355.38 ± 90.45	302.41 ± 131.76	350.44 ± 97.73	331.20 ± 120.57			
*Balance*								
UPST (sec)	I	4.60 ± 2.75	6.14 ± 3.38	7.08 ± 4.74	7.21 ± 4.95	0.879	0.545	0.725
	C	19.09 ± 18.36	34.93 ± 41.66	26.13 ± 28.39	38.85 ± 39.10			
**Mood status**								
*GDS-SF*	I	5.25 ± 2.77	4.75 ± 3.28	4.25 ± 3.28	4.50 ± 3.85	0.233	0.611	0.420
	C	2.75 ± 1.98	2.88 ± 2.17	2.00 ± 1.51	2.00 ± 2.88			
**Quality of life**								
*EQ5D-VAS*	I	64.04 ± 28.24	76.01 ± 23.42	68.59 ± 18.41	63.25 ± 30.34	0.364	0.215	0.436
	C	68.88 ± 23.69	70.75 ± 20.01	70.63 ± 17.50	67.38 ± 17.45			
*EQ5D-Utility*	I	0.70 ± 0.22	0.73 ± 0.24	0.82 ± 0.20	0.74 ± 0.33	0.823	0.555	0.552
	C	0.89 ± 0.16	0.81 ± 0.16	0.75 ± 0.24	0.77 ± 0.14			

I, intervention group; C, comparison group; SPMSQ, Short Portable Mental Status Questionnaire; IADL, Instrumental Activities of Daily Living Scale; SFT, senior functional test; TUG, timed up and go test; 6MWT, 6-min walk test; UPST timed unipedal stance test; GDS-SF, geriatric depression scale short form. EQ5D-VAS: The five domains of the Euroqol Health visual analogue scale (VAS). EQ-5D-Utility: The five domains of the Euroqol Health utility score. We used GEE to examine the main effect between the intervention group and the comparison group, as well as the time effect within baseline, 4th week, 8th week, and 12th week on the response of cognitive function, physical function, mood status, and quality of life. We also allow an interaction for group and time. Group *p* refers to the difference in the mean value regardless of the time points between groups, time *p* refers to the difference in four time points regardless of groups.

## Data Availability

Data are available on request due to restrictions (e.g., privacy or ethical). The data presented in this study are available on request from the corresponding author.

## Supplementary Materials

The following supporting information can be downloaded at: https://www.mdpi.com/article/10.3390/ijerph19063536/s1, Figure S1: One example of the interactive-video games “Step on Hamsters”. (**A**) A sensed pad was placed on the ground. (**B**) There were three levels of difficulty: Level 1 had two virtual holes, Level 2 had three virtual holes, and Level 3 had four virtual holes (Level 3 could be played with two participants and each watched two virtual holes). (**C**) The participant stepped on the sensed pad as fast as possible when virtual moles randomly appeared on the holes of the screen. (**D**) It showed the situation when played this interactive-video game. (**E**) It showed the final score that appeared on the screen after the game finished.
