# Supplementary material for: A Pilot Study of Interactive-Video Games in People with Mild Cognitive Impairment
Source: Int J Environ Res Public Health. 2022 Mar 16;19(6):3536. doi: 10.3390/ijerph19063536 (PMC8954200; doi:10.3390/ijerph19063536)
Supplement: Supplementary file 1 [file ijerph-19-03536-s001.zip › ijerph-1575186-supplementary.pdf]

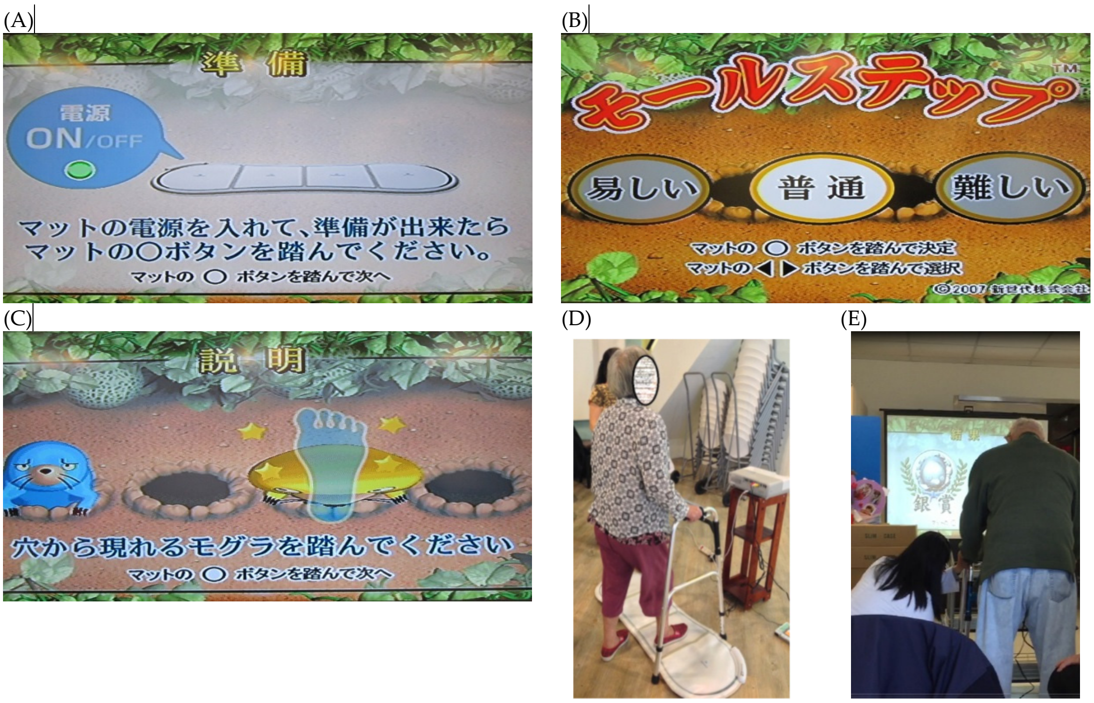

Figure S1. One example of the interactive-video games "Step on Hamsters". (**A**) A sensed pad was placed on the ground. (**B**) There were three levels of difficulty: Level 1 had two virtual holes, Level 2 had three virtual holes, and Level 3 had four virtual holes (Level 3 could be played with two participants and each watched two virtual holes). (**C**) The participant stepped on the sensed pad as fast as possible when virtual moles randomly appeared on the holes of the screen. (**D**) It showed the situation when played this interactive-video game. (**E**) It showed the final score that appeared on the screen after the game finished.
